# The phosphatase UBASH3B/Sts-1 is a negative regulator of Bcr-Abl kinase activity and leukemogenesis

**DOI:** 10.1038/s41375-019-0468-y

**Published:** 2019-04-08

**Authors:** Afsar A. Mian, Ines Baumann, Marcus Liebermann, Florian Grebien, Giulio Superti-Furga, Martin Ruthardt, Oliver G. Ottmann, Oliver Hantschel

**Affiliations:** 1 Department of Haematology, Division of Cancer and Genetics, School of Medicine, Cardiff University, Cardiff, UK; 2 CeMM Research Center for Molecular Medicine of the Austrian Academy of Sciences, Vienna, Austria; 3 Department of Hematology, Goethe University Frankfurt, Frankfurt/Main, Germany; 4 Center for Physiology and Pharmacology, Medical University of Vienna, Vienna, Austria; 5 Swiss Institute for Experimental Cancer Research, School of Life Sciences, École polytechnique fédérale de Lausanne, Lausanne, Switzerland; 6 Present Address: Center for Regenerative Medicine and Stem Cell Research, Aga Khan University, Karachi, Pakistan; 7 Present Address: Department of Pharmacology and Translational Research, Boehringer Ingelheim RCV GmbH & Co KG, Vienna, Austria; 8 Present Address: Institute for Medical Biochemistry, University of Veterinary Medicine Vienna, Vienna, Austria

**Keywords:** Chronic myeloid leukaemia, Targeted therapies, Oncogenes, Acute lymphocytic leukaemia

## To the Editor

The t(9;22) translocation results in the expression of the constitutively active BCR-ABL1 tyrosine kinase. It is detected in chronic myelogenous leukemia (CML) and in ~30% of adult acute lymphoblastic leukemia (ALL) patients [1]. Thus, Ph^+^ ALL is not only the largest genetically defined subgroup of ALL, but also characterized by a poor prognosis [2]. The two major protein isoforms of Bcr-Abl are p210 and p190. Whereas the shorter p190 isoform is specific for Ph^+^ ALL, the longer p210 isoform causes CML, but is also present in ~30% of Ph^+^ ALL patients [1, 3]. BCR-ABL1 was the first oncogene targeted successfully with the tyrosine kinase inhibitor (TKI) imatinib, which results in durable remissions in most CML patients and increased remission rates and survival in Ph^+^ ALL patients. Still, resistance to imatinib occurs particularly frequently in Ph^+^ ALL. Several next-generation TKIs were developed to address TKI resistance and intolerance [4]. Various TKI resistance mechanisms, including dozens of Bcr-Abl mutations, were described, but causes for resistance are still elusive in a significant portion of patients [5].

Deregulation of protein tyrosine phosphatases (PTP) plays an important role in maintaining a wide range of cancers. The ability of tyrosine phosphatases to antagonize oncogenic tyrosine kinases makes them candidate tumor suppressors. We previously showed that deregulation of PTP1B causes resistance in Ph^+^ leukemias [6]. The phosphatase Sts-1 (suppressor of T-cell receptor signaling 1, encoded by the human UBASH3B gene) was found to be transcriptionally upregulated in Ph^+^ ALL as compared with Ph^−^ ALL patients [7]. Notably, Sts-1 also is one of the most prominent interactors of Bcr-Abl as determined by a systematic interaction proteomics screen [8]. In two recent independent studies, Sts-1 was found to interact more strongly with the Bcr-Abl p210 isoform than with p190 and to be phosphorylated in Bcr-Abl expressing cells [9, 10]. Sts-1 and its only human and mouse paralogue, Sts-2 (UBASH3A), comprise an N-terminal ubiquitin-associated (UBA) domain, an Src homology 3 (SH3) domain and a C-terminal phosphoglycerate mutase (PGM) domain, which has structural homology with the histidine phosphatase superfamily. It was demonstrated that Sts-1 (and to a lesser extent Sts-2) possesses tyrosine phosphatase activity [11]. Strikingly, Sts-1 is a negative regulator of several tyrosine kinase pathways, including not only EGFR and PDGFR, but also ZAP-70 and SYK, thereby antagonizing T- and B-cell receptor signaling, respectively [12, 13]. As genetic and functional perturbation of kinase–phosphatase networks have been implicated in oncogenesis and based on our previous expression and proteomics data, we reasoned that the interaction between the Bcr-Abl kinase and the Sts-1 phosphatase may contribute to leukemogenesis. We therefore investigated the functional relationship between these two proteins, in particular the ability of Sts-1 to dephosphorylate Bcr-Abl and how it may contribute to TKI resistance in Ph^+^ ALL patients.

**Fig. 1:**
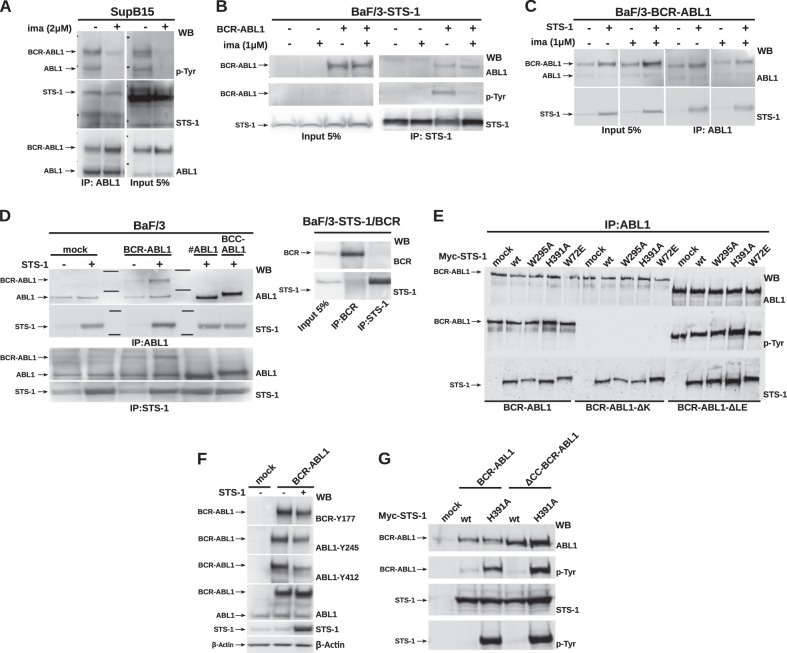
The Sts-1 phosphatase interacts with and dephosphorylates Bcr-Abl. **a** SupB15 was treated with imatinib (2 µM for 6 h). ABL1 immunoprecipitates (left panel) from total cell lysates and 5% input fraction (right panel) were immunoblotted with the indicated antibodies. **b** Ba/F3 cells transduced with STS-1 were additionally transduced with BCR-ABL1 and treated with imatinib (1 µM for 6 h). STS-1 immunoprecipitates (right panel) from total cell lysates and 5% input fraction (left panel) were immunoblotted with the indicated antibodies. **c** Ba/F3 cells transduced with BCR-ABL1 were additionally transduced with STS-1 and treated with imatinib (1 µM for 6 h). ABL1 immunoprecipitates (right panel) from total cell lysates and 5% input fraction (left panel) were immunoblotted with the indicated antibodies. **d** Ba/F3 cells transduced with full-length Bcr-Abl, the Abl-portion (#ABL1) only, the Bcr oligomerization domain directly fused to the Abl-portion (BCC-ABL1) or only the Bcr-portion (BCR) was additionally transduced with STS-1. ABL1 (upper panels) and STS-1 (lower panels) immunoprecipitates from total cell lysates were immunoblotted with the indicated antibodies. **e** HEK293 cells were cotransfected with BCR-ABL1 wt, Bcr-Abl kinase-dead (D382N; BCR-ABLΔK) or BCR-ABL1 lacking the C-terminal last exon region (BCR-ABLΔLE) with STS-1 wt and point mutations in functional domains (UBA (W72E), SH3 (W295A), and PGM (H391A)). ABL1 immunoprecipitates were analyzed by immunoblotting using the indicated antibodies. **f** Ba/F3 cells retrovirally expressing BCR-ABL1 were transfected with STS-1 or empty vector. Whole-cell lysates were then analyzed with the indicated antibodies. **g** HEK293 cells were cotransfected with BCR-ABL1 wt or an oligomerization-defective BCR-ABL1 lacking the coiled-coil domain (ΔCC-BCR-ABL1) with either STS-1 wt or a phosphatase-dead STS-1 mutant (H391A). Total cell lysates were analyzed by immunoblotting using the indicated antibodies

To study the interaction of Bcr-Abl p190 with Sts-1 and its dependence on kinase activity in Ph^+^ ALL cells, we performed coimmunoprecipitation (co-IP) assays of the endogenous p190 and Sts-1 in Sup-B15 cells either in the absence or presence of imatinib. Sts-1 binding to p190 was largely independent of the Bcr-Abl activation status, as their interaction was only mildly reduced in cells following exposure to imatinib (Fig. 1a). These data were confirmed by co-IP assays using murine Ba/F3 cells stably expressing p190 or Sts-1 as an independent cell-line model. p190 interacted with endogenous or overexpressed Sts-1 regardless of the activation status of Bcr-Abl (Fig. 1b, c).

In order to map the interaction mode of the two proteins, we first determined if Sts-1 binds to the Bcr- or Abl-portion of Bcr-Abl p190. These experiments were performed in Ba/F3 cells upon coexpression of Sts-1 with full-length Bcr-Abl, the Abl-portion (#ABL1) only, the Bcr oligomerization domain directly fused to the Abl-portion (BCC-ABL1) or only the Bcr-portion (BCR; Fig. 1d). These experiments showed that Sts-1 binding was mediated by the Abl-portion of Bcr-Abl, as all Abl-containing constructs, but not the Bcr-portion alone, bound Sts-1 (Fig. 1d). To map the Sts-1 domains that are required for binding, we performed co-IP assays using loss-of-function point mutations in all domains of Sts-1, including the UBA (W72E), SH3 (W295A), and PGM (H391A) domains (Fig. 1e). To further delineate the requirements for binding to Bcr-Abl, we also assayed these mutants in combination with Bcr-Abl mutants with either abolished kinase activity (ΔK; D382N mutation) or a deletion in the C-terminal last exon region (ΔLE). Sts-1 interacted with the N-terminal part of the Abl-portion of Bcr-Abl encompassing the SH3-SH2-kinase domain unit (Fig. 1e). This interaction was independent of the activation status and the various protein–protein interaction motifs in the C-terminal last exon region of Bcr-Abl. Furthermore, the interaction does not require a functional UBA-, SH3-, or PGM-domain of Sts-1 (Fig. 1e).

To study a possible functional interdependence of the Bcr-Abl/Sts-1 kinase–phosphatase interaction, we first investigated how Sts-1 may regulate Bcr-Abl kinase activity and autophosphorylation at different tyrosine (Y) residues: Y177 in the Bcr-portion is critical for Ras-MAPK signaling, whereas Y245 and Y412 in the Abl-portion are important markers for kinase activation [14]. Thus, we examined the autophosphorylation of Bcr-Abl in Ba/F3 cells in the presence and absence of Sts-1. In line with its binding properties, Sts-1 caused strong dephosphorylation of Bcr-Abl at Abl-Y245 and Abl-Y412, whereas Bcr-Y177 was only mildly dephosphorylated (Fig. 1f). In a second step, we cotransfected HEK293 cells with either Bcr-Abl or an oligomerization-deficient mutant (ΔCC-Bcr-Abl) together with either wild-type or a phosphatase-dead (H391A) Sts-1 [11]. We found that Sts-1 dephosphorylates both Bcr-Abl and itself, and that Sts-1 is a kinase substrate of Bcr-Abl. In fact, Sts-1 dephosphorylated Bcr-Abl and ΔCC-Bcr-Abl strongly and equally well (Fig. 1g). Conversely, only phosphatase-dead Sts-1, but not wild-type Sts-1, was strongly phosphorylated in the presence of Bcr-Abl, demonstrating that Sts-1 is able to dephosphorylate itself (Fig. 1g).

**Fig. 2:**
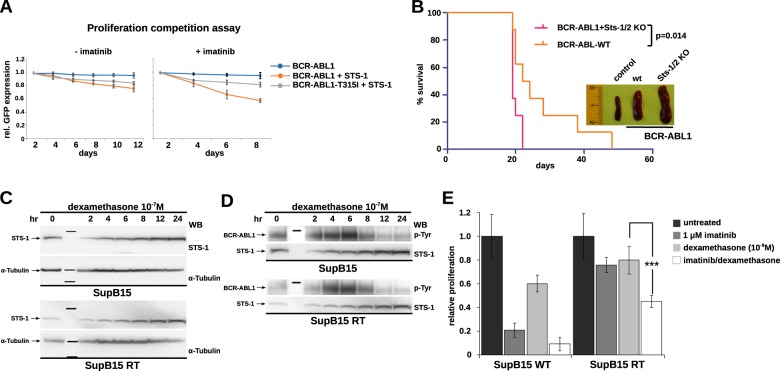
Sts-1 negatively regulates Bcr-Abl-dependent leukemogenesis and cell proliferation and its expression is upregulated by dexamethasone. **a** The proliferation competition assay with Ba/F3 cell transduced with BCR-ABL1 wt or T315I in the absence or presence of STS-1 expression and without (left) or with (right) imatinib treatment (2 µM). BCR-ABL1-positive cells are marked with GFP and the relative changes in GFP expression were measured by FACS and followed over time. Mean values ± SD from three replicates are shown.**b** Equal amounts of primary murine Lin^−^ c-Kit^+^ Sca-1^+^ cells from wt or Sts-1/Sts-2 knockout animals expressing BCR-ABL1 were injected in lethally irradiated recipient mice (*n* = 8 for each group). Overall survival of transplanted mice was monitored over 60 days. *P-*value = 0.014 was calculated using a logrank (Mantel–Cox) test. Representative spleens from control mice, BCR-ABL1/Sts-1/2 wt and BCR-ABL1/Sts-1/2 KO mice are shown. **c** SupB15 Ph^+^ ALL cells and their imatinib-resistant subline SupB15RT were exposed to 10^−7^ M dexamethasone, and STS-1 expression was investigated at the given time points. α-Tubulin was used as loading control. **d** SupB15 Ph^+^ ALL cells and their imatinib-resistant subline SupB15RT were exposed to 10^−7^M dexamethasone, and the effect of increasing expression of STS-1 on BCR-ABL1 phosphorylation was investigated by an anti-p-Tyr antibody. **e** SupB15 and SupB15RT were exposed to imatinib (1 µM) and 10^−7^M dexamethasone alone or in combination and proliferation was analyzed by the XTT assay. The bars represent the mean (±SEM) of three independent experiments, each performed in triplicates. Statistical significance was calculated using student’s *t* test. ^***^p ≤ 0.001

Given that Sts-1 may regulate kinase activity of Bcr-Abl by modulating its autophosphorylation, we next investigated whether Sts-1 impacts on cell proliferation in IL-3-independent Ba/F3 cells expressing wild-type (wt) Bcr-Abl or the gatekeeper mutation T315I, which conveys resistance to multiple TKIs. These cells were retrovirally transduced with GFP or Sts-1-GFP, and proliferation competition assays were performed over the course of 12 days. The expression of GFP alone did not alter the proliferation of BCR-ABL expressing Ba/F3 cells as revealed by the constant percentage of GFP positive cells (Fig. 2a). In contrast, expression of Sts-1-GFP reduced the proliferation of Ba/F3 cells expressing Bcr-Abl wt and T315I. Concomitant treatment with 1 µM imatinib further decreased proliferation of Bcr-Abl wt, but, as expected, not Bcr-Abl-T315I cells (Fig. 2a). These results indicate that Sts-1 activity negatively regulates cell proliferation induced by Bcr-Abl wt and T315I.

To assess the role of Sts-1 in Bcr-Abl-driven leukemogenesis, we examined the induction of Bcr-Abl p210-induced CML-like disease in wt vs. Sts-1/Sts-2 double-knockout bone marrow cells using a transduction/transplantation model. The absence of Sts-1/Sts-2 decreased the survival of recipient mice significantly and further aggravated the pronounced splenomegaly observed in mice transplanted with Bcr-Abl-expressing wt cells (Fig. 2b). These results indicate that Sts-1/Sts-2 are functionally relevant negative regulators of Bcr-Abl-dependent leukemogenesis in a CML mouse model.

The glucocorticoid dexamethasone and Sts-1 seem to regulate several common signaling pathways: Both inhibit T-cell receptor (TCR) signaling by regulating TCR expression and Sts-1 additionally inhibits certain downstream effectors [15]. In order to harness the therapeutic potential of Sts-1’s ability to inhibit growth of Bcr-Abl-positive cells, we explored whether dexamethasone may alter Sts-1 expression and activity. Exposure of  Sup-B15 and Sup-B15RT cells to clinically relevant concentrations of dexamethasone increased Sts-1 expression over time, accompanied by decreased Bcr-Abl autophosphorylation in Sup-B15 cells (Fig. 2c, d), indicating that it increases the sensitivity of Bcr-Abl-transformed cells to TKIs. In cell proliferation assays, concomitant treatment with dexamethasone and imatinib showed stronger inhibition than each drug alone in both Sup-B15 and Sup-B15RT cells (Fig. 2e).

Deregulation of the tyrosine phosphatase Sts-1 may be an important and pharmacologically targetable mechanism for Bcr-Abl mutation-independent resistance. Upregulation of Sts-1 in Ph^+^ ALL together with its direct interaction with both p190 and p210 Bcr-Abl strongly suggests a functional deregulation of protein phosphorylation similar to that we previously showed for PTP1B [6]. The fact that its normal function can be restored by ectopic overexpression not only indicates a central role for Sts-1 in the regulation of Bcr-Abl but also that the deregulation of Sts-1 is based on a loss of balance between Bcr-Abl kinase and Sts-1 phosphatase activity. This establishes the upregulation of Sts-1 by drugs, such as dexamethasone, as a valid therapeutic approach for increasing the sensitivity to TKIs.

In conclusion, we delineated the molecular interaction mode of the Sts-1 phosphatase with the Bcr-Abl kinase and provide strong evidence that Sts-1 is a negative regulator of Bcr-Abl signaling, cell proliferation, and leukemogenesis. In addition, the parallel study by Udainiya et al. (cosubmitted) shows a broad impact of Sts-1 on the Bcr-Abl phosphoproteome network and precisely delineated the Sts-1 interactome using quantitative functional proteomics techniques. Furthermore, we show that modulation of Sts-1 expression by dexamethasone influences TKI sensitivity of Ph^+^ ALL cells. Therefore, the inclusion of dexamethasone for therapy regimens in Ph^+^ ALL for may increase sensitivity to TKIs by upregulating Sts-1.

## Supplementary information

Supplemental Material

## References

[1] Deininger MW, Goldman JM, Melo JV (2000). The molecular biology of chronic myeloid leukemia. Blood.

[2] Ottmann O. G., Pfeifer H. (2009). Management of Philadelphia chromosome-positive acute lymphoblastic leukemia (Ph+ ALL). Hematology.

[3] Reckel S, Gehin C, Tardivon D, Georgeon S, Kukenshoner T, Lohr F (2017). Structural and functional dissection of the DH and PH domains of oncogenic Bcr-Abl tyrosine kinase. Nat Commun.

[4] Hantschel O, Grebien F, Superti-Furga G (2012). The growing arsenal of ATP-competitive and allosteric inhibitors of BCR-ABL. Cancer Res.

[5] O’Hare T, Zabriskie MS, Eiring AM, Deininger MW (2012). Pushing the limits of targeted therapy in chronic myeloid leukaemia. Nat Rev Cancer.

[6] Koyama N, Koschmieder S, Tyagi S, Portero-Robles I, Chromic J, Myloch S (2006). Inhibition of phosphotyrosine phosphatase 1B causes resistance in BCR-ABL-positive leukemia cells to the ABL kinase inhibitor STI571. Clin Cancer Res.

[7] Juric D, Lacayo NJ, Ramsey MC, Racevskis J, Wiernik PH, Rowe JM (2007). Differential gene expression patterns and interaction networks in BCR-ABL-positive and -negative adult acute lymphoblastic leukemias. J Clin Oncol.

[8] Brehme M, Hantschel O, Colinge J, Kaupe I, Planyavsky M, Kocher T (2009). Charting the molecular network of the drug target Bcr-Abl. Proc Natl Acad Sci USA.

[9] Reckel S, Hamelin R, Georgeon S, Armand F, Jolliet Q, Chiappe D (2017). Differential signaling networks of Bcr-Abl p210 and p190 kinases in leukemia cells defined by functional proteomics. Leukemia.

[10] Cutler JA, Tahir R, Sreenivasamurthy SK, Mitchell C, Renuse S, Nirujogi RS (2017). Differential signaling through p190 and p210 BCR-ABL fusion proteins revealed by interactome and phosphoproteome analysis. Leukemia.

[11] Mikhailik A, Ford B, Keller J, Chen Y, Nassar N, Carpino N (2007). A phosphatase activity of Sts-1 contributes to the suppression of TCR signaling. Mol Cell.

[12] Carpino N, Turner S, Mekala D, Takahashi Y, Zang H, Geiger TL (2004). Regulation of ZAP-70 activation and TCR signaling by two related proteins, Sts-1 and Sts-2. Immunity.

[13] Raguz J, Wagner S, Dikic I, Hoeller D (2007). Suppressor of T-cell receptor signalling 1 and 2 differentially regulate endocytosis and signalling of receptor tyrosine kinases. FEBS Lett.

[14] Hantschel O (2012). Structure, regulation, signaling, and targeting of abl kinases in cancer. Genes Cancer.

[15] Migliorati G, Bartoli A, Nocentini G, Ronchetti S, Moraca R, Riccardi C (1997). Effect of dexamethasone on T-cell receptor/CD3 expression. Mol Cell Biochem.

