# Supplementary material for: The phosphatase UBASH3B/Sts-1 is a negative regulator of Bcr-Abl kinase activity and leukemogenesis
Source: Leukemia. 2019 Apr 8;33(9):2319–23. doi: 10.1038/s41375-019-0468-y (PMC6756289; doi:10.1038/s41375-019-0468-y)
Supplement: Supplementary file 1 — Supplemental Material [file 41375_2019_468_MOESM1_ESM.pdf]

Supplementary Information

The phosphatase UBASH3B/Sts-1 is a negative regulator of Bcr-Abl kinase activity and leukemogenesis

Running title: UBASH3B/Sts-1 is a negative regulator of Bcr-Abl

Afsar A. Mian, Ines Baumann, Marcus Liebermann, Florian Grebien, Giulio Superti-Furga, Martin Ruthardt, Oliver G. Ottmann and Oliver Hantschel

Content

Materials and Methods

Materials and Methods

Plasmids

The cDNAs encoding p190$^{BCR/ABL}$, p190$^{BCR/ABL\ T315I}$, p210$^{BCR/ABL}$ and the various Bcr-Abl deletion mutants have been previously described[1]. All retroviral expression vectors were based on the bi-cistronic vector PAULO[1]. The coding sequence of STS-1 was obtained by PCR using a pcDNA3-Sts-1 plasmid as the template[2]. Point mutations were introduced using the Quickchange site-directed mutagenesis kit (Stratagene).

Cell lines, transient transfection and retroviral infection

All human and mouse cell lines were obtained from the German Collection of Microorganisms and Cell Cultures (DSMZ, Braunschweig, Germany). Tyrosine kinase inhibitor-resistant Sup-B15 (Sup-B15 RT) cells were generated and maintained as previously described[3]. Transfection and retroviral infection were performed as previously described[1,4].

Antibodies and immunoblotting

Antibodies were purchased at Cell Signaling Technology (pAbl(Y245) #2868, pAbl(Y412), pBcr(Y177) #3901), Rockland Immunochemicals (Sts-1 #600-401-870) or prepared in-house and purified on a ProteinG affinity resin (Abl clone 24-21, pY clone 4G10).

The immunoblot signals were measured with the Odyssey imager (Li-Cor) and quantified using the Image Studio Software (Li-Cor) or using the FluorChem 2 digital

imaging system and quantified by densitometric analysis using AlphaEase® FC Software (Version 6.0.2).

Co-Immunoprecipitation

0.4-1 mg total protein of cell lysates were used for co-immunoprecipitation experiments. After preclearing with 20 μl of an equilibrated Immunosorb A Sepharose slurry (Medicago AB, Uppsala Sweden), 3 μl of α-ABL or α-Sts-1 antibody, in addition to 20 μl of protein-A Sepharose, were used to precipitate Bcr-Abl or Sts-1 from the lysates. Co-immunoprecipitated proteins were detected by SDS-PAGE and immunoblotting using the appropriate antibodies. The immunoblot and co-immunoprecipitation results shown in figures 1 and 2 are representative images from three independent repeats.

Mouse bone-marrow transplantation assay

Total mouse bone marrow cells from wt or Sts1/2 knock-out animals (*Mus musculus*, B6129SF1, female, 6-12 weeks) were transduced with pMSCV-IRES-GFP-based retroviral vectors for 72 hours in the presence of IL-3, IL-6, SCF and 7 μg/ml polybrene as previously described4. Transduced cells were injected into lethally irradiated (10 Gy) wt recipients via the tail vein. Upon signs of sickness, mice were sacrificed analyzed as previously described[4]. During all animal experiments we adhered to the 3 R principles (reduction, replacement, and refinement). All animal experiments were performed according to ethical animal license protocols approved by the authorities of the Austrian government.

Proliferation competition assays

Ba/F3 cells were infected with PAULO vectors harboring p190$^{BCR\text{-}ABL\ wt}$ or p190$^{BCR\text{-}ABL\ T315I}$ and the pinco Sts-1 or the pinco vector alone. IL-3 was removed from the media of Ba/F3- infected cells by washing them twice with phosphate-buffered saline (PBS) and then continuously maintained in the absence of IL-3. Proliferation and competition between single- and double-infected cell fractions were monitored by FACS analysis of GFP expression in transfected Ba/F3 cells.

References

1. Beissert T, Hundertmark A, Kaburova V, Travaglini L, Mian AA, Nervi C*, et al.* Targeting of the N-terminal coiled coil oligomerization interface by a helix-2 peptide inhibits unmutated and imatinib-resistant BCR/ABL. *Int J Cancer* 2008; **122:** 2744-2752.

2. Raguz J, Wagner S, Dikic I, Hoeller D. Suppressor of T-cell receptor signalling 1 and 2 differentially regulate endocytosis and signalling of receptor tyrosine kinases. *FEBS Lett* 2007; **581:** 4767-4772.

3. Koyama N, Koschmieder S, Tyagi S, Portero-Robles I, Chromic J, Myloch S*, et al.* Inhibition of phosphotyrosine phosphatase 1B causes resistance in BCR-ABL-positive leukemia cells to the ABL kinase inhibitor STI571. *Clin Cancer Res* 2006; **12:** 2025-2031.

4. Grebien F, Hantschel O, Wojcik J, Kaupe I, Kovacic B, Wyrzucki AM*, et al.* Targeting the SH2-Kinase Interface in Bcr-Abl Inhibits Leukemogenesis. *Cell* 2011; **147:** 306-319.
